# Avacopan is effective in inducing remission for MPA/GPA, regardless of changes in serum C5a levels: a single-center study in Japan

**DOI:** 10.1186/s41927-025-00555-2

**Published:** 2025-08-11

**Authors:** Yusuke Ushio, Hiromi Shimada, Risa Wakiya, Shusaku Nakashima, Taichi Miyagi, Koichi Sugihara, Rina Mino, Mao Mizusaki, Kanako Chujo, Naoto Manabe, Norimitsu Kadowaki, Hiroaki Dobashi

**Affiliations:** Division of Hematology, Faculty of Medicine, Rheumatology and Respiratory Medicine, Department of Internal Medicine, Kagawa University, 1750-1 Ikenobe, Miki-cho, Kita-gun, Kagawa, 761-0793 Japan

**Keywords:** Avacopan, Microscopic polyangiitis, Granulomatosis with polyangiitis, Remission induction therapy, Serum C5a

## Abstract

**Background:**

Avacopan, a selective oral C5a receptor antagonist, was approved for the treatment of microscopic polyangiitis (MPA) and granulomatosis with polyangiitis (GPA) in 2021. However, there are still limited reports on its efficacy and safety in real-world settings, specifically regarding its impact on the Vasculitis Damage Index (VDI), and its effects on serum biomarkers are poorly understood. This study aimed to evaluate the efficacy and safety of avacopan in remission induction therapy for MPA/GPA in a real-world setting, as well as its effect on serum C5a levels.

**Methods:**

This retrospective study investigated patients with MPA/GPA who received remission induction therapy with a 6-month follow-up at our institution, comparing those who received avacopan with those who did not. Efficacy and safety were evaluated by comparing the remission rate, changes in Birmingham Vasculitis Activity Score (BVAS) and VDI score after 6 months, daily glucocorticoid (GC) dose, and incidence of adverse events (AEs). Changes in serum C5a levels, measured using ELISA, were compared between both groups at baseline and 3 months.

**Results:**

A total of 66 patients with MPA/GPA were included, with 14 and 52 patients in the avacopan and non-avacopan groups, respectively. The remission rate and decrease in BVAS was comparable between both groups. However, those who received avacopan had a significantly smaller increase in VDI score, significantly lower daily GC dose at 1, 3, and 6 months, and significantly lower incidence of GC-related AEs within 6 months. Serum C5a levels did not significantly change in the avacopan group but significantly decreased in the non-avacopan group. Remission was achieved in the avacopan group regardless of whether serum C5a decreased or increased.

**Conclusions:**

Treatment with avacopan appears to effectively suppress the increase in VDI score, enable reduced GC dosage, and lower the incidence of GC-related AEs during remission induction therapy for MPA/GPA in a real-world setting. Furthermore, avacopan may suppress disease activity regardless of serum C5a levels.

**Clinical trial number:**

Not applicable.

**Supplementary Information:**

The online version contains supplementary material available at 10.1186/s41927-025-00555-2.

## Background

Anti-neutrophil cytoplasmic antibody (ANCA)-associated vasculitis (AAV) is characterized by the development of systemic small vessel vasculitis alongside ANCA production. AAV includes three diseases entities: microscopic polyangiitis (MPA), granulomatosis with polyangiitis (GPA), and eosinophilic granulomatosis with polyangiitis (EGPA) [1]. AAV can lead to severe complications such as rapidly progressive glomerulonephritis, interstitial lung disease, alveolar hemorrhage, and mononeuritis multiplex [2–5], as well as irreversible organ damage due to the toxicity associated with the use of glucocorticoids (GCs), which are key in the treatment of AAV [6, 7].

Avacopan, a selective oral C5a receptor antagonist, was approved as an adjunctive treatment for MPA and GPA in several countries, including Japan, in 2021. The phase III ADVOCATE trial revealed that avacopan was noninferior to prednisone in achieving remission at 26 weeks and superior in sustaining remission at 52 weeks [8]. This positions avacopan as a promising alternative to GC in the treatment of MPA and GPA, potentially reducing GC-associated toxicity. However, reports on the efficacy and safety of avacopan for MPA and GPA in real-world settings are still limited, particularly regarding its impact on the Vasculitis Damage Index (VDI) score [9]. Furthermore, the effects of avacopan on serum biomarkers remain unclear. To address this knowledge gap, this study aimed to evaluate the efficacy and safety of avacopan in remission induction therapy for MPA and GPA in a real-world setting, as well as its effects on serum C5a levels, which is a biomarker of AAV [10–13].

## Methods

### Study population

This retrospective study included patients with MPA or GPA who received remission induction therapy with GC plus rituximab (RTX) or cyclophosphamide (CY) at Kagawa University Hospital in Japan between January 2017 and December 2023 and were followed up for 6 months. MPA and GPA were diagnosed based on the 2022 American College of Rheumatology (ACR)/European League Against Rheumatism (EULAR) classification criteria for MPA [14] and GPA [15]. Patients were divided into two groups: the avacopan group (avacopan + remission induction therapy) and the non-avacopan group (remission induction therapy only).

## Remission induction therapy

Remission induction therapy with either RTX or CY was selected at the discretion of the treating physician. RTX was administered at a dose of 375 mg/m² once weekly for 4 consecutive weeks, while CY was given at 15 mg/kg per dose every 2–3 weeks. The number and timing of doses were adjusted based on individual patient characteristics, including age, renal function, and clinical course. These regimens were generally consistent with those used in the ADVOCATE trial [8]. GC tapering was also performed at the physician’s discretion based on the patient’s disease activity and response to therapy. Regarding remission maintenance therapy, no patients received RTX within the 6-month observation period. However, at our institution, RTX is administered on demand after 6 months upon the reappearance of peripheral CD19-positive B cells (defined as ≥ 1 cell/µL), using an individually tailored regimen similar to that of the MAINRITSAN2 trial [16]. Azathioprine was administered in some patients during the observation period. Generally, the choice of maintenance therapy was at the discretion of the attending physician.

## Data collection

The following data were collected from the patients’ medical records at the start of remission induction therapy (i.e., baseline): demographic information (age, sex), disease status (newly diagnosed or relapsed), ANCA status (anti-proteinase 3 [PR3] positive or anti-myeloperoxidase [MPO] positive), type of vasculitis (MPA or GPA), the type of remission induction therapy (RTX or CY), and the number of days from baseline to the initiation of avacopan (in the avacopan group only). The following data were collected from baseline to 6 months: Birmingham Vasculitis Activity Score (BVAS) [17], VDI score [9], daily GC dose (prednisolone-equivalent), remission maintenance therapy (azathioprine or RTX), and adverse events (AEs).

## Efficacy

The differences in remission rate and changes in BVAS, VDI score, and daily GC dose from baseline to 6 months were compared between groups. Remission was defined as a BVAS of 0. Patients who relapsed, defined as reappearance of disease activity with BVAS > 0 and requiring an increase in GC or immunosuppressive agents, and those who died were considered to have no remission at the subsequent observation points.

## Safety

The incidence of AEs from baseline to 6 months were compared between groups. Based on the EULAR search criteria [18] and Glucocorticoid Toxicity Index (GTI) [19], the following were defined as potentially associated with GC toxicity (i.e., GC-related AEs): hypertension, diabetes mellitus, osteoporosis, spinal compression fractures, serious infections (Grade 3 or higher, according to the National Cancer Institute Common Terminology Criteria for Adverse Events (version 5.0)), cataracts, and glaucoma.

### Serum C5a analysis

Serum C5a levels at baseline and at 3 months were compared between groups, only including those with complete data. The collected serum samples were stored at − 80 °C until analysis to preserve stability, and measurements were made using ELISA kits (ab193695, Abcam, UK) according to the manufacturers’ instructions.

### Statistical analysis

Data are expressed as the median (interquartile range) or number (%). Differences between the two groups were analyzed using the Wilcoxon rank sum test for continuous variables and Fisher’s exact test for categorical variables. Differences between groups at baseline and at 1, 3, and 6 months were analyzed using the Wilcoxon signed rank test. All p-values were two-sided, with *p* < 0.05 considered statistically significant. All analyses were conducted using the JMP^Ⓡ^ Pro 14 software (SAS Institute, Cary, USA).

## Results

### Patient characteristics

The study cohort included 66 patients with MPA/GPA, with 14 and 52 patients in the avacopan and non-avacopan groups, respectively. The baseline patient characteristics are shown in Table 1, with no significant differences in age, sex, disease status, ANCA status, type of vasculitis, BVAS, and VDI score between the two groups. In both groups, more than 90% of the patients were MPO-ANCA positive, while none were PR3-ANCA positive. Regarding remission induction therapy, RTX was more frequently used in the avacopan group, but this was not statistically significant. All patients were treated with GCs, and the daily GC dose at baseline was similar in both groups. Regarding the year of treatment initiation, the avacopan group had a significantly greater proportion of patients who received remission induction therapy in 2021 or later, because avacopan was approved in Japan in 2021. For remission maintenance therapy, azathioprine was administered to 2 of 14 patients in the avacopan group (both following CY induction), and to 25 of 52 patients in the non-avacopan group (16 following CY and 9 following RTX). No patients received RTX for maintenance therapy (Table 1).

**Table 1:** Patient characteristics

	Avacopan group (*N* = 14)	Non-avacopan group (*N* = 52)	*p*
Age, years	73.0 (68.0–81.5)	75.0 (69.3–80.0)	0.666
Sex, female, n (%)	7 (50.0)	40 (76.9)	0.092
Newly diagnosed, n (%)	9 (64.3)	39 (75.0)	0.503
Relapsed, n (%)	5 (35.7)	13 (25.0)	0.503
ANCA status			
PR3-ANCA positive, n (%)	0 (0)	0 (0)	1.000
MPO-ANCA positive, n (%)	13 (92.9)	48 (92.3)	1.000
Negative, n (%)	1 (7.1)	4 (7.7)	1.000
Type of vasculitis			
GPA, n (%)	3 (21.4)	15 (28.9)	0.742
MPA, n (%)	11 (78.6)	37 (71.2)	0.742
Birmingham Vasculitis Activity Score (BVAS)	10.0 (8.0–17.3)	13.0 (8.0–18.8)	0.470
Organ involvement (BVAS ≥ 1) †			
General	11 (78.6)	34 (65.4)	0.520
Chest	7 (50.0)	21 (40.4)	0.555
Renal	6 (42.9)	35 (67.3)	0.124
Nervous system	5 (35.7)	13 (25.0)	0.503
Ear, nose, and throat	3 (21.4)	7 (13.5)	0.431
Mucous membranes or eyes	0 (0)	5 (9.6)	0.576
Vasculitis Damage Index (VDI) score	0 (0–2.0)	0 (0–0)	0.255
Year remission induction therapy was initiated			
In 2021 or later	14 (100.0)	14 (26.9)	< 0.001**
Remission induction therapy			
Intravenous RTX, n (%)	12 (85.7)	30 (57.7)	0.066
Intravenous CY, n (%)	2 (14.3)	22 (42.3)	0.066
Use of any GC, n (%)	14 (100.0)	52 (100.0)	-
Intravenous GC pulse, n (%) ‡	1 (7.1)	6 (11.5)	1.000
Daily GC dose (prednisolone-equivalent) at baseline, mg/day	40.0 (30.0–52.5)	43.5 (38.5–50.0)	0.668
Days from baseline to the introduction of avacopan, day	13.5 (8.8–19.0)	-	-
Remission maintenance therapy			
Azathioprine after CY induction, n (%)	2 (14.3)	16 (30.8)	0.318
Azathioprine after RTX induction, n (%)	0 (0)	9 (17.3)	0.186
RTX (within 6 months), n (%) §	0 (0)	0 (0)	-

Data are presented as median (IQR) or as n (%), unless otherwise indicated. ANCA, anti-neutrophil cytoplasmic antibody; PR3, anti-proteinase 3; MPO, anti-myeloperoxidase; GPA, granulomatosis with polyangiitis; MPA, microscopic polyangiitis; BVAS, Birmingham Vasculitis Activity Score; VDI, Vasculitis Damage Index; RTX, rituximab; CY, cyclophosphamide; GC, glucocorticoid

For statistical analyses, *p < 0.05, **p < 0.01. *P*-value: Wilcoxon rank sum test, Fisher’s exact test

† Organ involvement was based on BVAS ≥ 1

‡ An infusion of methylprednisolone for 3 consecutive days at a dose of 500 or 1000 mg per day

§ Rituximab was not administered as maintenance therapy during the 6-month observation period. It was administered on demand after 6 months based on the reappearance of peripheral CD19-positive B cells

### Comparison of treatment efficacy with and without avacopan

**Fig. 1:**
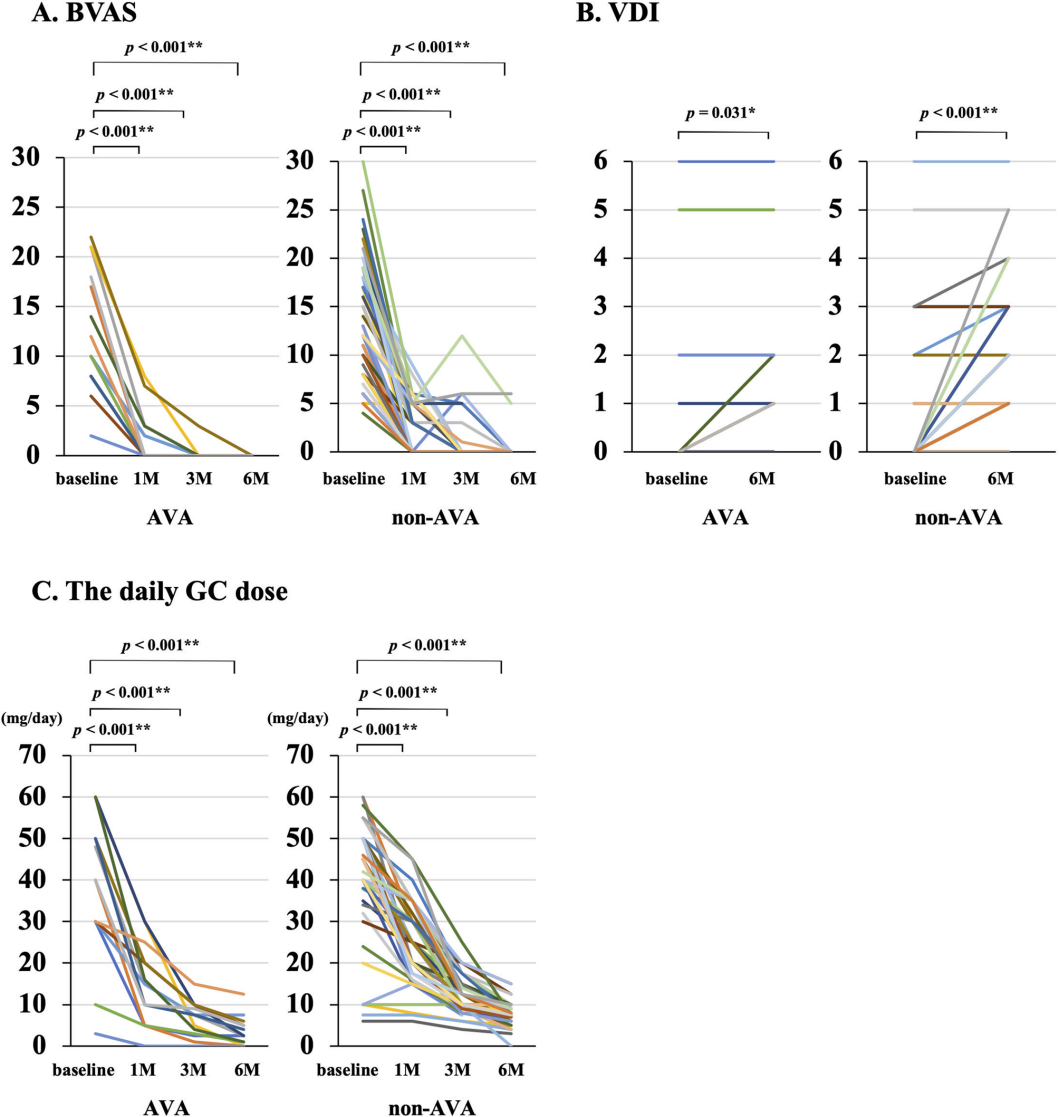
Changes in BVAS, VDI score, and daily GC dose from baseline to 6 months. (**a**) BVAS, (**b**) VDI score, and (**c**) daily GC dose. BVAS, Birmingham Vasculitis Activity Score; VDI, Vasculitis Damage Index; GC, glucocorticoid; AVA, avacopan; non-AVA, non-avacopan. For statistical analyses, *p < 0.05, **p < 0.01. *P*-value: Wilcoxon signed rank test

**Fig. 2:**
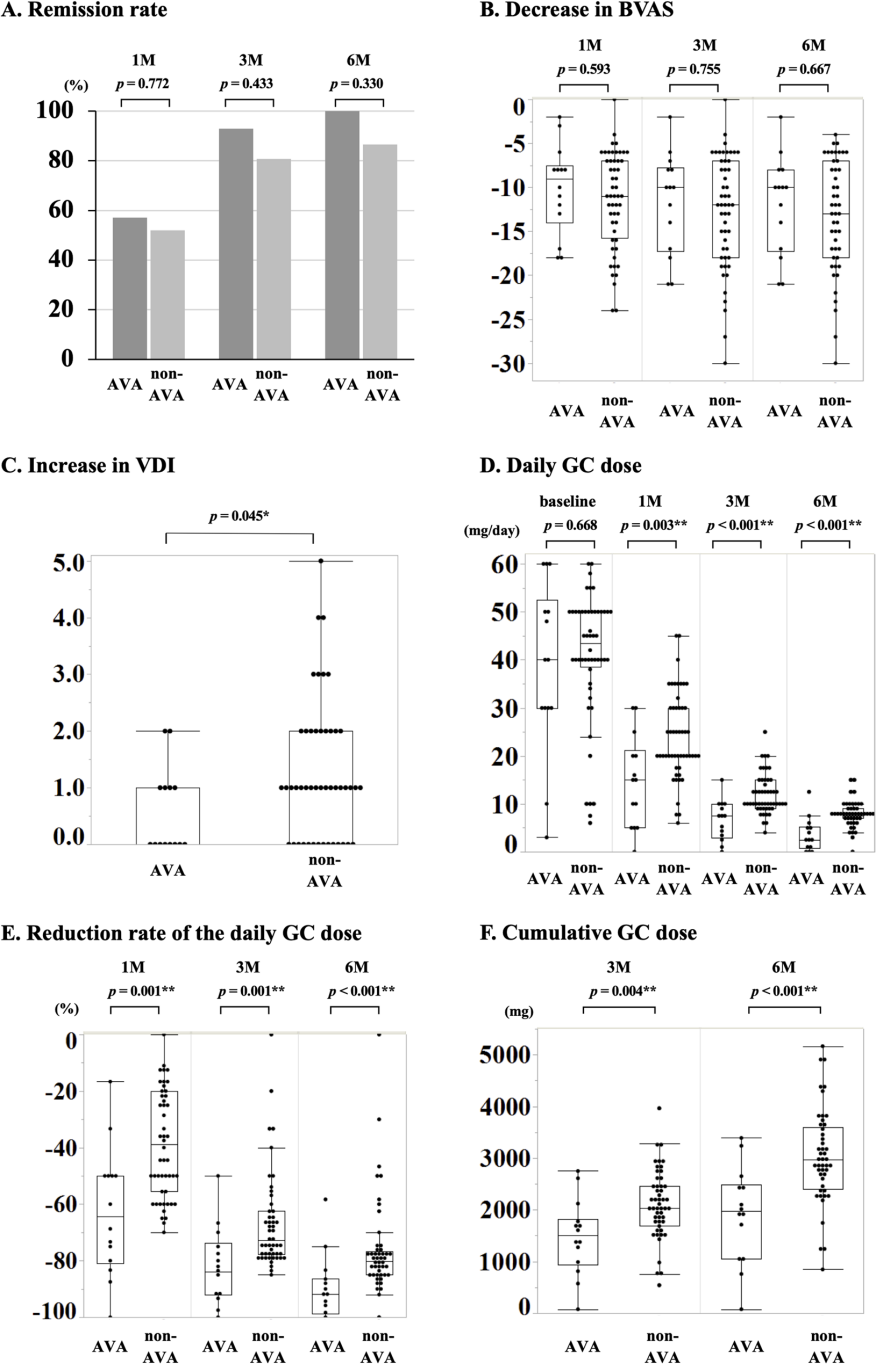
Differences in treatment efficacy between the avacopan and non-avacopan groups. (**a**) Remission rate. (**b**) Decrease in BVAS. (**c**) Increase in VDI score. (**d**) Daily GC dose. (**e**) Reduced daily GC dose. (**f**) Cumulative GC dose. BVAS, Birmingham Vasculitis Activity Score; VDI, Vasculitis Damage Index; GC, glucocorticoid; AVA, avacopan; non-AVA, non-avacopan. For statistical analyses, *p < 0.05, **p < 0.01. *P*-value: Wilcoxon rank sum test, Fisher’s exact test

Patient outcomes are shown in Additional file 1. BVAS at 1, 3, and 6 months, respectively, was 0 [0–3.0], 0 [0–0], and 0 [0–0] in the avacopan group and 0 [0–5.0], 0 [0–0], and 0 [0–0] in the non-avacopan group, demonstrating a significant decrease from baseline to all observation points in both groups (Fig. 1a). Meanwhile, the remission rates and change in BVAS were not significantly different between both groups at all time- points. Comparing the avacopan versus non-avacopan groups, remission rates were 57.1% vs. 51.9% at 1 month, 92.9% vs. 80.8% at 3 months, and 100.0% vs. 86.5% at 6 months (Fig. 2a), whereas the changes in BVAS from baseline were − 9.0 [− 14.0–−7.5] vs. −11.0 [− 15.8–−7.0] at 1 month, − 10.0 [− 17.3–−7.8] vs. −12.0 [− 18.0–−7.0] at 3 months, and − 10.0 [− 17.3–−8.0] vs. −13.0 [− 18.0–−7.0] at 6 months (Fig. 2b).

Compared to baseline, the VDI scores of the avacopan and non-avacopan groups significantly increased at 6 months (1.0 [0.8–2.0] and 2.0 [1.0–3.0], respectively; Fig. 1b). However, this increase in VDI score was significantly smaller in the avacopan group (0 [0–1.0] vs. 1.0 [0–2.0]; Fig. 2c). The details regarding VDI items with increased scores are shown in Table 2. Although there were no statistically significant differences in individual VDI items between the two groups, a trend toward an increase in VDI score in items potentially related to GC toxicity (i.e., vertebral collapse, cataract, hypertension, and diabetes) was observed in the non-avacopan group.

**Table 2:** Details of the vasculitis damage index items with increased scores

VDI items with increased scores	Avacopan group (*N* = 14)	Non-avacopan group (*N* = 52)	*p*
Musculoskeletal			
Osteoporosis/vertebral collapse, n (%)	0 (0)	2 (3.9)	1.000
Osteomyelitis, n (%)	0 (0)	1 (1.9)	1.000
Ocular			
Cataract, n (%)	0 (0)	2 (3.9)	1.000
ENT			
Hearing loss, n (%)	1 (7.1)	1 (1.9)	0.382
Pulmonary			
Pulmonary fibrosis, n (%)	1 (7.1)	7 (13.5)	1.000
Cardiovascular			
Diastolic BP > 95 or requiring antihypertensive therapy, n (%)	1 (7.1)	12 (23.1)	0.270
Peripheral vascular disease			
Complicated venous thrombosis, n (%)	0 (0)	2 (3.9)	1.000
Renal			
Estimated/measured GFR < 50%, n (%)	2 (14.3)	11 (21.2)	0.718
Proteinuria ≥ 0.5 g/24 hr, n (%)	0 (0)	3 (5.8)	1.000
Neuropsychiatric			
Cerebrovascular accident, n (%)	0 (0)	1 (1.9)	1.000
Peripheral neuropathy, n (%)	3 (21.4)	8 (15.4)	0.688
Other			
Diabetes, n (%)	0 (0)	10 (19.2)	0.104

Data are expressed as n (%). VDI, Vasculitis Damage Index; ENT, Ear, nose, and throat; BP, blood pressure; GFR, glomerular filtration rate

For statistical analyses, *p < 0.05, **p < 0.01. *P*-value: Fisher’s exact test

The daily GC doses at 1, 3, and 6 months were 15.0 [5.0–21.3], 7.5 [2.9–10.0], and 2.5 [0.8–5.3] mg/day in the avacopan group and 20.0 [20.0–30.0], 10.0 [9.0–15.0], and 7.5 [7.0–9.0] mg/day in the non-avacopan group, showing significant decreases from baseline to all observation points in both groups (Fig. 1c). Significantly lower GC doses at 1, 3, and 6 months were seen in the avacopan group (Fig. 2d). Moreover, the avacopan group had a significantly greater decrease in daily GC dose from baseline to 1, 3, and 6 months versus the non-avacopan group at all-time points (− 64.4% [− 80.8%–−50.0%] vs. −38.8% [− 55.6%–−20.0%] at 1 month, − 83.9% [− 92.1%–−73.8%] vs. −72.8% [− 77.8%–−62.5%] at 3 months, and − 91.8% [− 98.8%–−86.5%] vs. −80.3% [− 85.0%–−76.7%] at 6 months; Fig. 2e). The cumulative GC dose (except for pulse dose) until 3 and 6 months was significantly lower in the avacopan group versus the non-avacopan group (1501.0 [944.9–1823.8] vs. 2030.0 [1696.0–2454.5] mg at 3 months, 1978.3 [1053.4–2485.0] vs. 2972.5 [2401.5–3599.0] mg at 6 months; Fig. 2f). Similarly, the cumulative GC dose, including the pulse dose, was significantly low in the avacopan group (1501.0 [944.9–2242.5] vs. 2183.8 [1717.5–2738.0] mg at 3 months, and 2051.3 [1053.4–2796.9] vs. 3072.5 [2401.5–3634.0] mg at 6 months (Additional file 2).

### Safety of avacopan

The incidence of any AE was comparable between both groups. However, GC-related AEs (e.g., hypertension, diabetes mellitus, osteoporosis, spinal compression fractures, serious infections, cataracts, and glaucoma) were significantly less frequent in the avacopan group (7.1% vs. 50.0%). Notably, drug-induced liver injury due to avacopan occurred in 4 patients (28.6%), leading to discontinuation in 2 patients (14.3%) and dose reduction in 2 patients (14.3%) (Table 3). All 4 patients showed improvement in liver function after discontinuation or dose reduction. The clinical characteristics and courses of these four patients are summarized in Additional file 3.

**Table 3:** Adverse events within 6 months after treatment

Adverse events	Avacopan group (*N* = 14)	Non-avacopan group (*N* = 52)	*p*
Any adverse event, n (%)	7 (50.0)	36 (69.2)	0.215
Any infection, n (%)	5 (35.7)	21 (40.4)	1.000
Any potentially glucocorticoid-related adverse events, n (%)	1 (7.1)	26 (50.0)	0.005^**^
Number of potentially glucocorticoid-related adverse events	1	34	-
Hypertension, n (%)	1 (7.1)	14 (26.9)	0.161
Serious infection, n (%)	0 (0)	6 (11.5)	0.328
Diabetes mellitus, n (%)	0 (0)	10 (19.2)	0.104
Osteoporosis, Spinal compression fracture, n (%)	0 (0)	2 (3.9)	1.000
Cataract, Glaucoma, n (%)	0 (0)	2 (3.9)	1.000
Death, n (%)	0 (0)	2 (3.9)	1.000
Death due to infection, n (%)	0 (0)	1 (1.9)	1.000
Death due to vasculitis worsening, n (%)	0 (0)	1 (1.9)	1.000
Relapse of vasculitis, n (%)	0 (0)	3 (5.8)	1.000
Drug-induced liver injury due to avacopan, n (%)	4 (28.6)	-	-
Discontinuation of avacopan, n (%)	2 (14.3)	-	-
Dose reduction of avacopan, n (%)	2 (14.3)	-	-

Data are expressed as n (%)

For statistical analyses, *p < 0.05, **p < 0.01. *P*-value: Wilcoxon rank sum test

### Effect of avacopan on serum C5a levels

**Fig. 3:**
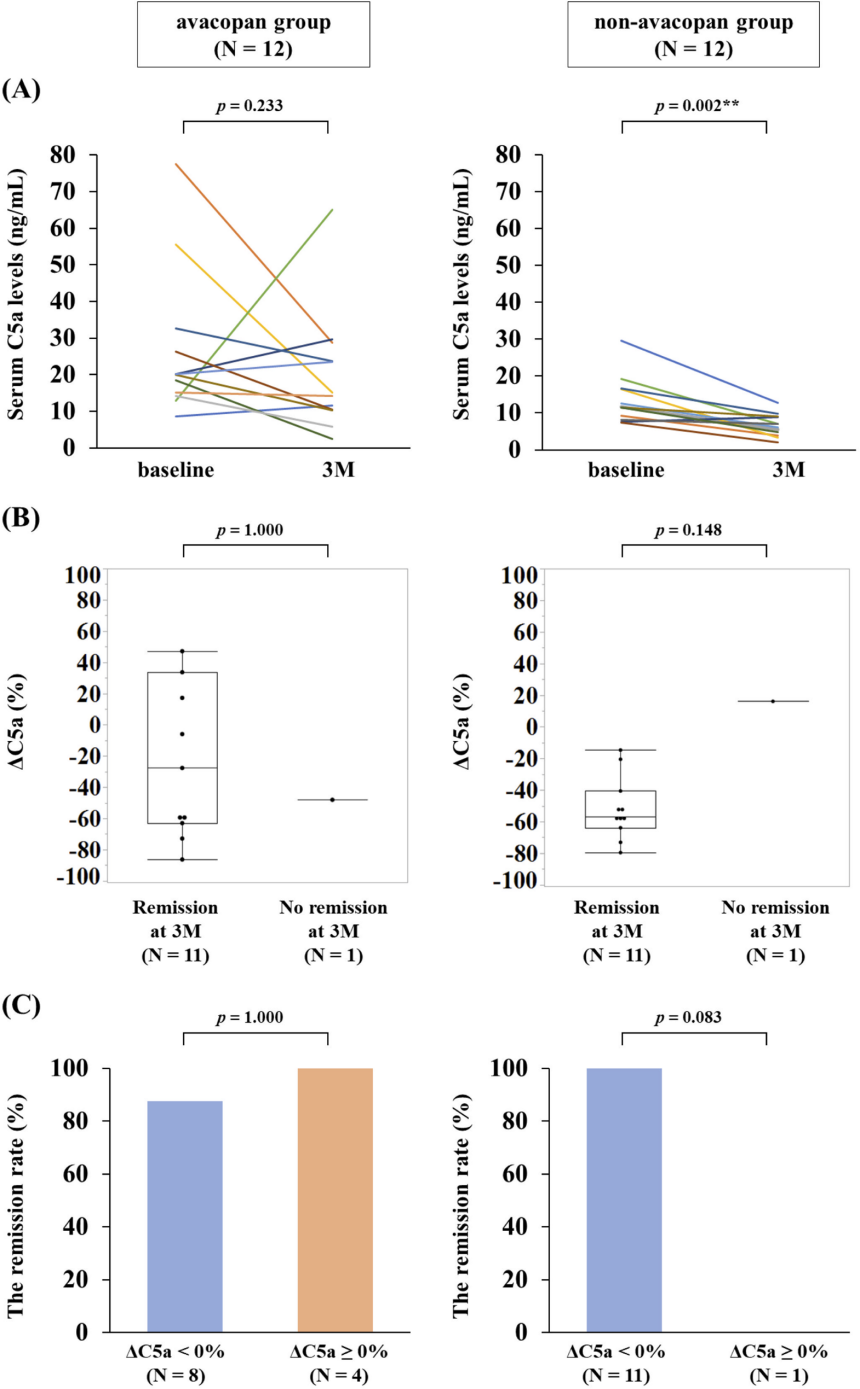
Comparison of changes in serum C5a levels between the avacopan and non-avacopan groups. (**a**) Serum C5a levels at baseline and at 3 months. (**b**) Differences in the percentage change in serum C5a levels from baseline to 3 months (ΔC5a) between patients who achieved remission at 3 months versus those who did not. (**c**) Differences in remission rates between patients with ΔC5a < 0% versus ΔC5a ≥ 0%. Remission was defined as BVAS = 0. One patient in the avacopan group, with a ΔC5a of 405.1% who achieved remission, is not shown in Fig. 3b, as this value exceeds the scale. For statistical analyses, *p < 0.05, **p < 0.01. *P*-value: Wilcoxon signed rank test, Wilcoxon rank sum test

Serum C5a levels were measured in 12/14 patients in the avacopan group and 12/52 patients in the non-avacopan group. Patient characteristics of this subgroup are presented in Supplementary Table 2. In the avacopan group, serum C5a levels tended to decrease from baseline (20.0 [14.5–31.1] ng/mL) to 3 months (14.6 [10.4–27.4] ng/mL), although this was not significant. In the non-avacopan group, serum C5a levels significantly decreased from baseline (11.6 [8.4–16.6] ng/mL) to 3 months (6.5 [4.1–9.0] ng/mL) (Fig. 3a). The percentage change in serum C5a levels (ΔC5a, %) from baseline to 3 months is shown in Fig. 3b. Remission at 3 months was achieved in 11/12 patients in each group. Patients who achieved remission had comparable ΔC5a to those that did not. In the avacopan group, 7/8 patients (87.5%) with ΔC5a < 0% and 4/4 patients (100%) with ΔC5a ≥ 0% achieved remission, while 1 patient with ΔC5a < 0% did not achieve remission. In the non-avacopan group, 11/11 patients (100%) with ΔC5a < 0% achieved remission, while 1 patient with ΔC5a ≥ 0% did not achieve remission (Fig. 3c).

## Discussion

This study investigated the efficacy and safety of avacopan in a real-world setting by comparing patients who received remission induction therapy with versus without avacopan.

Regarding efficacy, both groups demonstrated a rapid decrease in BVAS after treatment with no significant differences in remission rates, suggesting that both treatments were comparable. Notably, the daily GC dose at 1, 3, and 6 months from baseline, as well as the cumulative GC dose until 3 and 6 months, were all significantly lower in the avacopan group. These results align with previous clinical trial data and highlight the GC-sparing potential of avacopan in real-world settings. Importantly, the increase in VDI scores over six months—a surrogate for early cumulative organ damage—was significantly smaller in the avacopan group. Although there were no significant differences in individual VDI items between the two groups, the use of avacopan appeared to inhibit the increase in VDI scores in items associated with GC toxicity, particularly hypertension and diabetes (Table 2). Given that VDI scores typically increase early in AAV, with approximately 80% of patients having an increase in at least 1 item by 6 months [7], early use of avacopan may play a critical role in preventing irreversible organ damage.

While the incidence of AE was similar between the two groups, GC-related AEs were significantly reduced in the avacopan group, underscoring the benefit of rapid GC tapering facilitated by avacopan. Notably, 28.6% of patients in the avacopan group developed drug-induced liver injury—a frequency consistent with the Japanese subanalysis of the ADVOCATE trial (27.3%) [20]. However, this incidence is notably higher than the rate of serious AEs related to liver function abnormalities in the overall ADVOCATE trial population (5.4%) [8]. Furthermore, observational studies from Japan have reported a broader range of liver injury incidence (16.7–38.1%) [21]– [22], indicating the potentially higher susceptibility of Japanese patients. Several cases of vanishing bile duct syndrome have also been reported in Japan [23]– [24], raising the possibility that racial or genetic differences may contribute to the susceptibility to avacopan-induced hepatotoxicity. These observations emphasize the importance of careful liver function monitoring during the first 6 months of avacopan therapy, despite its favorable profile in reducing GC-associated toxicity.

A novel feature of our study is the examination of serum C5a levels before and after treatment. C5a is known to play a critical role in the pathogenesis of AAV [25, 26]. C5a induces the expression of endothelial selectins, which facilitates the interaction between neutrophils and endothelial cells, thereby promoting neutrophil rolling, intravascular crawling, and transcellular migration [27]. Additionally, C5a can translocate MPO, PR3, and other neutrophil granule proteins to the neutrophil cell surface, where ANCA can bind to them [28]. The binding of ANCA to these antigens activates neutrophils and induces the release of the neutrophil extracellular traps, which causes inflammatory damage to the endothelium [29]. Previous studies have demonstrated markedly elevated C5a levels in the peripheral blood of patients with active AAV versus those with inactive AAV [10–12], suggesting its potential as biomarker for disease activity in AAV. However, no previous studies have assessed whether avacopan affects circulating C5a levels.

In the present study, serum C5a levels significantly decreased in the non-avacopan group but did not change significantly in the avacopan group (Fig. 3a). This suggests that avacopan had no direct effect on reducing serum C5a levels. Interestingly, in the non-avacopan group, all patients with decreased C5a levels achieved remission, while the only patient with increased serum C5a levels did not, consistent with previous findings that serum C5a levels may reflect disease activity [10–13]. Conversely, most patients in the avacopan group achieved remission regardless of whether serum C5a levels were increased or decreased (Fig. 3c). This discrepancy could be explained by the unique mechanism of action of avacopan. Avacopan is thought to control vasculitis through inhibiting neutrophil activation by blocking the interaction between C5a and C5a receptors, thereby potentially suppressing disease activity without necessarily reducing circulating C5a levels.

Although elevated serum C5a levels may be associated with relapse risk [10–13], increased C5a levels during avacopan treatment may not necessarily indicate impending relapse. Thus, alternative or complementary biomarkers are needed to monitor treatment response and predict relapse in patients receiving avacopan.

The potential role of baseline serum C5a levels in guiding precision medicine with avacopan is also an important consideration. Given avacopan’s mechanism of action, patients with higher baseline serum C5a levels may benefit more from the treatment. However, the predictive value of baseline serum C5a as a marker for avacopan responsiveness warrants further investigation.

In our study, baseline serum C5a levels were higher in the avacopan group. However, baseline BVAS, organ involvement, baseline GC dose, and the use of other induction therapies were comparable between the groups. Given the small sample size, we could not fully clarify the relationship between baseline serum C5a levels and BVAS. Elucidating the associations among baseline serum C5a levels, disease activity, and treatment response may facilitate the development of biomarker-based personalized strategies for avacopan. Future studies in larger cohorts are needed to explore this possibility.

Finally, the pattern variations of C5a changes between groups may reflect the combined influence of the pharmacological mechanism of avacopan and GC exposure. Although baseline characteristics were comparable between groups, both the daily GC dose at 3 months and the cumulative GC dose up to 3 months were lower in the avacopan group (Additional file 4). Furthermore, patients with increased C5a levels tended to have received lower cumulative GC doses up to 3 months (Additional files 5, 6), suggesting that GC exposure may contribute to reductions in serum C5a.

This study has several limitations. First, biases could have been introduced based on the year that the participants received treatment. Since avacopan was approved in Japan in 2021, the avacopan group included more patients who received remission induction therapy in 2021 or later compared to the non-avacopan group. This temporal difference may have influenced therapeutic decisions, particularly GC tapering strategies, as recent trials such as PEXIVAS and LoVAS [30, 31] have promoted reduced-dose regimens. Second, although more patients in the avacopan group received RTX, the difference was not statistically significant and may reflect evolving treatment trends. Third, the study did not include patients with PR3-ANCA-positive MPA or GPA—a population less common in Japan [32–34]—limiting generalizability to Western cohorts. Finally, serum C5a levels were measured in a very small number of patients, and the observed changes could have been influenced not only by avacopan, but also by GC and RTX/CY administration.

Despite these limitations, this study provides valuable insights. To our knowledge, it is the first observational study to evaluate the impact of avacopan on both VDI and serum C5a levels in a real-world setting. While recent studies with larger cohorts have investigated the effectiveness and safety of avacopan [35–37], most have focused solely on patients treated with avacopan, without a comparison group. In this context, our study offers a unique perspective by comparing patients treated with and without avacopan, focusing on novel and clinically meaningful outcomes. Further prospective studies with larger sample sizes and longer follow-up are needed to validate these findings.

## Conclusions

In this retrospective study, avacopan treatment appeared to effectively suppress the increase in VDI, reduce GC dosage, and lower the incidence of GC-related AEs in remission induction therapy for MPA/GPA in a real-world setting. Furthermore, avacopan may suppress disease activity regardless of serum C5a levels.

## Supplementary Information

Below is the link to the electronic supplementary material.

Supplementary Material 1

Supplementary Material 2

Supplementary Material 3

Supplementary Material 4

Supplementary Material 5

Supplementary Material 6

## Data Availability

The datasets used and/or analyzed during the current study are available from the corresponding author upon reasonable request.

## Abbreviations

ANCA: Anti-neutrophil cytoplasmic antibody
AAV ANCA: Associated vasculitis
MPA: Microscopic
GPA: Granulomatosis with polyangiitis
EGPA: Eosinophilic granulomatosis with polyangiitis
GC: Glucocorticoid
VDI: Vasculitis Damage Index
RTX: Rituximab
CY: Cyclophosphamide
ACR: American College of Rheumatology
EULAR: European League Against Rheumatism
PR3: Anti-proteinase 3
MPO: Anti-myeloperoxidase
BVAS: Birmingham Vasculitis Activity Score
AE: Adverse event
GTI: Glucocorticoid Toxicity Index
