# Supplementary figures and images for: Avacopan is effective in inducing remission for MPA/GPA, regardless of changes in serum C5a levels: a single-center study in Japan
Source: BMC Rheumatol. 2025 Aug 11;9:99. doi: 10.1186/s41927-025-00555-2 (PMC12337394; doi:10.1186/s41927-025-00555-2)

Supplementary Figure 1. Patient disposition.


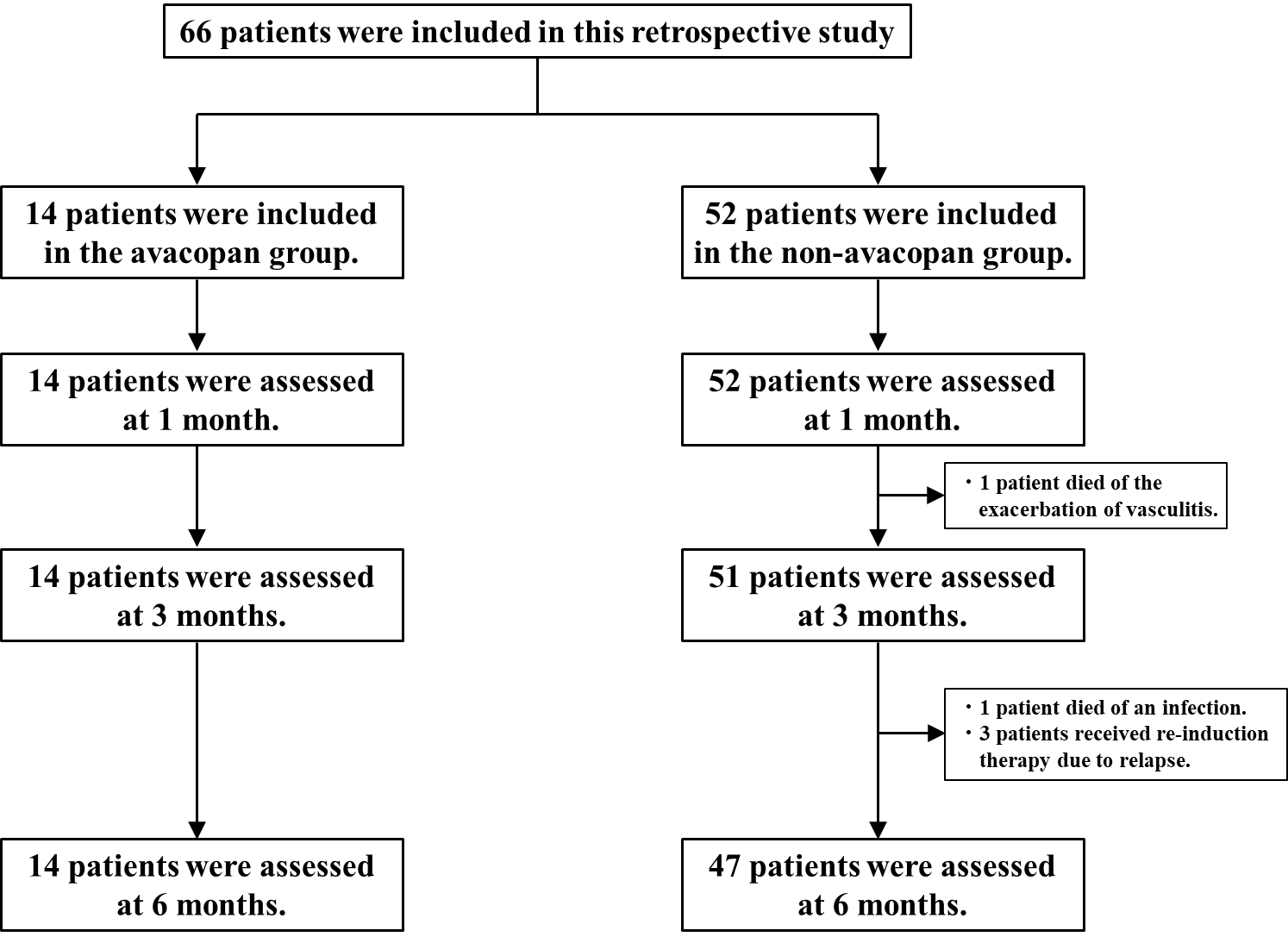

Supplement: Supplementary file 1 — Supplementary Material 1 [file 41927_2025_555_MOESM1_ESM.docx]
