# Supplementary material for: Avacopan is effective in inducing remission for MPA/GPA, regardless of changes in serum C5a levels: a single-center study in Japan
Source: BMC Rheumatol. 2025 Aug 11;9:99. doi: 10.1186/s41927-025-00555-2 (PMC12337394; doi:10.1186/s41927-025-00555-2)
Supplement: Supplementary file 2 — Supplementary Material 2 [file 41927_2025_555_MOESM2_ESM.docx]

Supplementary Figure 2. Cumulative GC dose (including pulse therapy) at 3 and 6 months.


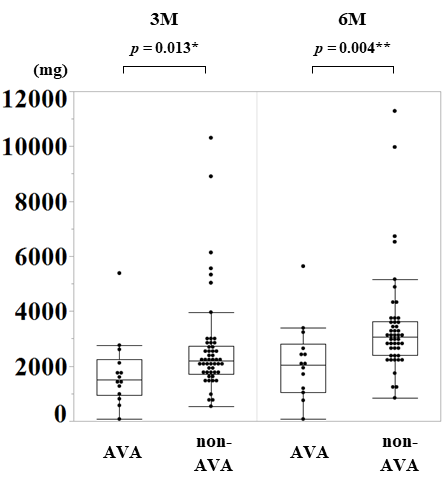


GC, glucocorticoid; AVA, avacopan; non-AVA, non-avacopan

For statistical analyses, *p < 0.05, **p < 0.01. P-value: Wilcoxon rank sum test
