# Supplementary material for: Avacopan is effective in inducing remission for MPA/GPA, regardless of changes in serum C5a levels: a single-center study in Japan
Source: BMC Rheumatol. 2025 Aug 11;9:99. doi: 10.1186/s41927-025-00555-2 (PMC12337394; doi:10.1186/s41927-025-00555-2)
Supplement: Supplementary file 3 — Supplementary Material 3 [file 41927_2025_555_MOESM3_ESM.docx]

Supplementary Table 1. Clinical characteristics and courses of the four patients who developed avacopan-induced liver injury.

|  | Patient 1 | Patient 2 | Patient 3 | Patient 4 |
| --- | --- | --- | --- | --- |
| Age, years | 73 | 77 | 84 | 84 |
| Sex | Female | Male | Male | Female |
| Type of vasculitis | GPA | MPA | GPA | MPA |
| Time from onset of liver injury from avacopan initiation, days | 99 | 76 | 75 | 39 |
| Peak AST (IU/mL) (normal: 13–30) | 38 | 944 | 32 | 49 |
| Peak ALT (IU/mL) (normal: 7–23) | 85 | 1399 | 84 | 94 |
| Peak ALP (IU/mL) (normal: 38–113) | 327 | 136 | 145 | 94 |
| Peak γ-GT (IU/mL) (normal: 9–32) | 511 | 737 | 177 | 230 |
| Peak TSB (mg/dL) (normal: 0.4–1.5) | 0.4 | 2.6 | 0.6 | 0.7 |
| Management | Dose reduction | Discontinuation | Dose reduction | Discontinuation |
| Outcome | Resolution | Resolution | Resolution | Resolution |

AST: aspartate aminotransferase; ALT: alanine aminotransferase; ALP: alkaline phosphatase; γ-GT: gamma-glutamyltransferase; TSB: total serum bilirubin.
