# Supplementary material for: Avacopan is effective in inducing remission for MPA/GPA, regardless of changes in serum C5a levels: a single-center study in Japan
Source: BMC Rheumatol. 2025 Aug 11;9:99. doi: 10.1186/s41927-025-00555-2 (PMC12337394; doi:10.1186/s41927-025-00555-2)
Supplement: Supplementary file 4 — Supplementary Material 4 [file 41927_2025_555_MOESM4_ESM.docx]

Supplementary Table 2. Characteristics of patients included in the serum C5a analysis.

|  | Avacopan group （N=12） | Non-avacopan group （N=12） | *p* |
| --- | --- | --- | --- |
| At baseline |  |  |  |
| Age, years | 73.0 (69.5–82.5) | 76.5 (71.0–79.8) | 0.685 |
| Sex, female, n (%) | 7 (58.3) | 10 (83.3) | 0.371 |
| Newly diagnosed, n (%) | 8 (66.7) | 8 (66.7) | 1.000 |
| Relapsed, n (%) | 4 (33.3) | 4 (33.3) | 1.000 |
| ANCA status | | | |
| PR3-ANCA positive, n (%) | 0 (0) | 0 (0) | 1.000 |
| MPO-ANCA positive, n (%) | 11 (91.7) | 12 (100.0) | 1.000 |
| Negative, n (%) | 1 (8.3) | 0 (0) | 1.000 |
| Type of vasculitis | | | |
| GPA, n (%) | 3 (25.0) | 5 (41.7) | 0.667 |
| MPA, n (%) | 9 (75.0) | 7 (58.3) | 0.667 |
| Birmingham Vasculitis Activity Score (BVAS) | 10.0  (8.0–16.3) | 11.5  (7.3–18.5) | 0.622 |
| Organ involvement (BVAS ≥ 1) † | | | |
| General | 10 (83.3) | 10 (83.3) | 1.000 |
| Chest | 6 (50.0) | 3 (25.0) | 0.400 |
| Renal | 4 (33.3) | 8 (66.7) | 0.220 |
| Nervous system | 5 (41.7) | 5 (41.7) | 1.000 |
| Ear, nose, and throat | 3 (25.0) | 2 (16.7) | 1.000 |
| Mucous membranes or eyes | 0 (0) | 1 (8.3) | 1.000 |
| Vasculitis Damage Index (VDI) score | 0 (0–1.8) | 0 (0–0.8) | 0.517 |
| Remission induction therapy | | | |
| Intravenous RTX, n (%) | 10 (83.3) | 11 (91.7) | 1.000 |
| Intravenous CY, n (%) | 2 (16.7) | 1 (8.3) | 1.000 |
| Use of any GC, n (%) | 12 (100.0) | 12 (100.0) | - |
| Intravenous GC pulse, n (%) ‡ | 1 (8.3) | 1 (8.3) | 1.000 |
| Daily GC dose (prednisolone-equivalent) at baseline, mg/day | 40.0 (30.0–57.5) | 42.5 (36.3–50.0) | 1.000 |
| Days from baseline to the introduction of avacopan, day | 14.5 (11.0–19.0) | - | - |
| Year in which remission induction therapy was initiated | | | |
| In 2021 or later | 12 (100.0) | 0 (0) | < 0.001** |
| At 1 month |  |  |  |
| Daily GC dose (prednisolone-equivalent), mg/day | 13.0 (5.0–23.8) | 20.0 (16.3–20.0) | 0.258 |
| %Change in the daily GC dose from baseline to 1 month, % | −66.7 (−82.5–−50.0) | −52.8 (−60.0–−31.4) | 0.097 |
| At 3 months |  |  |  |
| Daily GC dose (prednisolone-equivalent) at 3 months, mg/day | 6.3 (2.6–10.0) | 10.0 (9.3–12.5) | 0.020* |
| %Change in the daily GC dose from baseline to 3 months, % | −84.2 (−92.9–−71.9) | −76.3 (−79.4–−61.1) | 0.021* |
| Cumulative GC dose (prednisolone-equivalent) up to 3 months, mg | 1571 (794–1935) | 1855 (1630–2122) | 0.141 |
| Serum C5a analysis |  |  |  |
| Serum C5a levels at baseline, ng/mL | 20.0 (14.5–31.1) | 11.6 (8.4–16.6) | 0.010* |
| Serum C5a levels at 3 months, ng/mL | 14.6 (10.4–27.4) | 6.5 (4.1–9.0) | 0.004** |
| Percentage change in serum C5a levels from baseline to 3 months (ΔC5a), % | −37.9 (−62.3–29.5) | −55.0 (−62.6–−25.5) | 0.403 |

Data are presented as median (IQR) or as n (%), unless otherwise indicated. ANCA, anti-neutrophil cytoplasmic antibody; PR3, anti-proteinase 3; MPO, anti-myeloperoxidase; GPA, granulomatosis with polyangiitis; MPA, microscopic polyangiitis; BVAS, Birmingham Vasculitis Activity Score; VDI, Vasculitis Damage Index; RTX, rituximab; CY, cyclophosphamide; GC, glucocorticoid

For statistical analyses, **p* < 0.05, ***p* < 0.01. *P*-value: Wilcoxon rank sum test, Fisher’s exact test

† Organ involvement was based on BVAS ≥ 1.

‡ An infusion of methylprednisolone for 3 consecutive days at a dose of 500 or 1000 mg per day.
