# Supplementary material for: Avacopan is effective in inducing remission for MPA/GPA, regardless of changes in serum C5a levels: a single-center study in Japan
Source: BMC Rheumatol. 2025 Aug 11;9:99. doi: 10.1186/s41927-025-00555-2 (PMC12337394; doi:10.1186/s41927-025-00555-2)
Supplement: Supplementary file 5 — Supplementary Material 5 [file 41927_2025_555_MOESM5_ESM.docx]

Supplementary Table 3. Comparison of patient characteristics in the avacopan group based on changes in serum C5a levels.

|  | Decrease in serum C5a (ΔC5a < 0%) (N = 8) | Increase in serum C5a  (ΔC5a ≥ 0%)  (N = 4) | *p* |
| --- | --- | --- | --- |
| At baseline |  |  |  |
| Age, years | 73.0 (66.8–79.0) | 77.0 (69.5–83.8) | 0.732 |
| Sex, female, n (%) | 6 (75.0) | 1 (25.0) | 0.222 |
| Newly diagnosed, n (%) | 8 (100.0) | 0 (0) | 0.002^**^ |
| Relapsed, n (%) | 0 (0) | 4 (100.0) | 0.002^**^ |
| ANCA status | | | |
| PR3-ANCA positive, n (%) | 0 (0) | 0 (0) | 1.000 |
| MPO-ANCA positive, n (%) | 8 (100.0) | 3 (75.0) | 0.333 |
| Negative, n (%) | 0 (0) | 1 (25.0) | 0.333 |
| Type of vasculitis | | | |
| GPA, n (%) | 2 (25.0) | 1 (25.0) | 1.000 |
| MPA, n (%) | 6 (75.0) | 3 (75.0) | 1.000 |
| Birmingham Vasculitis Activity Score (BVAS) | 13.0 (8.5–17.8) | 9.0 (3.5–10.0) | 0.123 |
| Organ involvement (BVAS ≥ 1) † | | | |
| General | 8 (100.0) | 2 (50.0) | 0.091 |
| Chest | 5 (62.5) | 1 (25.0) | 0.546 |
| Renal | 2 (25.0) | 2 (50.0) | 0.548 |
| Nervous system | 5 (62.5) | 0 (0) | 0.081 |
| Ear, nose, and throat | 3 (37.5) | 0 (0) | 0.491 |
| Mucous membranes or eyes | 0 (0) | 0 (0) | - |
| Vasculitis Damage Index (VDI) score | 0 (0–0) | 3.0 (1.3–4.8) | 0.002^**^ |
| Remission induction therapy | | | |
| Intravenous RTX, n (%) | 6 (75.0) | 4 (100.0) | 0.515 |
| Intravenous CY, n (%) | 2 (25.0) | 0 (0) | 0.515 |
| Use of any GC, n (%) | 8 (100.0) | 4 (100.0) | - |
| Intravenous GC pulse, n (%) ‡ | 1 (12.5) | 0 (0) | 1.000 |
| Daily GC dose (prednisolone-equivalent) at baseline, mg/day | 45.0 (32.5–57.5) | 20.0 (4.8–52.5) | 0.195 |
| Days from baseline to the introduction of avacopan, day | 15.5 (13.3–19.0) | 11.0 (9.5–20.8) | 0.443 |
| Year in which remission induction therapy was initiated | | | |
| In 2021 or later | 8 (100.0) | 4 (100.0) | - |
| At 1 month |  |  |  |
| Daily GC dose (prednisolone-equivalent), mg/day | 18.0 (10.0–23.8) | 5.0 (1.3–23.8) | 0.229 |
| %Change in the daily GC dose from baseline to 1 month, % | −66.7 (−78.8–−37.5) | −66.7 (−95.8–−50.0) | 0.549 |
| At 3 months |  |  |  |
| Daily GC dose (prednisolone-equivalent) at 3 months, mg/day | 8.3 (4.3–10.0) | 2.8 (0.6–8.3) | 0.200 |
| %Change in the daily GC dose from baseline to 3 months, % | −39.5 (−51.9–−22.8) | −17.3 (−44.4–−4.0) | 0.203 |
| Cumulative GC dose (prednisolone-equivalent) up to 3 months, mg | 1615 (1375–1935) | 618 (139–2178) | 0.203 |

Data are presented as median (IQR) or as n (%), unless otherwise indicated. ANCA, anti-neutrophil cytoplasmic antibody; PR3, anti-proteinase 3; MPO, anti-myeloperoxidase; GPA, granulomatosis with polyangiitis; MPA, microscopic polyangiitis; BVAS, Birmingham Vasculitis Activity Score; VDI, Vasculitis Damage Index; RTX, rituximab; CY, cyclophosphamide; GC, glucocorticoid

For statistical analyses, **p* < 0.05, ***p* < 0.01. *P*-value: Wilcoxon rank sum test, Fisher’s exact test

† Organ involvement was based on BVAS ≥ 1.

‡ An infusion of methylprednisolone for 3 consecutive days at a dose of 500 or 1000 mg per day.
