# Supplementary material for: Avacopan is effective in inducing remission for MPA/GPA, regardless of changes in serum C5a levels: a single-center study in Japan
Source: BMC Rheumatol. 2025 Aug 11;9:99. doi: 10.1186/s41927-025-00555-2 (PMC12337394; doi:10.1186/s41927-025-00555-2)
Supplement: Supplementary file 6 — Supplementary Material 6 [file 41927_2025_555_MOESM6_ESM.docx]

Supplementary Table 4. Comparison of patient characteristics in the non-avacopan group based on changes in serum C5a levels.

|  | Decrease in serum C5a (ΔC5a < 0%)  (N = 11) | Increase in serum C5a  (ΔC5a ≥ 0%)  (N = 1) | *p* |
| --- | --- | --- | --- |
| At baseline |  |  |  |
| Age, years | 77.0 (71.0–80.0) | 76.0 (76.0–76.0) | 1.000 |
| Sex, female, n (%) | 9 (81.8) | 1 (100.0) | 1.000 |
| Newly diagnosed, n (%) | 7 (63.6) | 1 (100.0) | 1.000 |
| Relapsed, n (%) | 4 (36.4) | 0 (0) | 1.000 |
| ANCA status | | | |
| PR3-ANCA positive, n (%) | 0 (0) | 0 (0) | - |
| MPO-ANCA positive, n (%) | 11 (100.0) | 1 (100.0) | - |
| Negative, n (%) | 0 (0) | (0) | - |
| Type of vasculitis | | | |
| GPA, n (%) | 4 (36.4) | 1 (100.0) | 0.417 |
| MPA, n (%) | 7 (63.6) | 0 (0) | 0.417 |
| Birmingham Vasculitis Activity Score (BVAS) | 12.0 (7.0–19.0) | 11.0 (11.0–11.0) | 0.884 |
| Organ involvement (BVAS ≥ 1) † | | | |
| General | 10 (90.9) | 0 (0) | 0.167 |
| Chest | 3 (27.3) | 0 (0) | 1.000 |
| Renal | 7 (63.6) | 1 (100.0) | 1.000 |
| Nervous system | 5 (45.5) | 0 (0) | 1.000 |
| Ear, nose, and throat | 1 (9.1) | 1 (100.0) | 0.167 |
| Mucous membranes or eyes | 1 (9.1) | 0 (0) | 1.000 |
| Vasculitis Damage Index (VDI) score | 0 (0–1.0) | 0 (0–0) | 0.703 |
| Remission induction therapy | | | |
| Intravenous RTX, n (%) | 10 (90.9) | 1 (100.0) | 1.000 |
| Intravenous CY, n (%) | 1 (9.1) | 0 (0) | 1.000 |
| Use of any GC, n (%) | 11 (100.0) | 1 (100.0) | - |
| Intravenous GC pulse, n (%) ‡ | 1 (9.1) | 0 (0) | 1.000 |
| Daily GC dose (prednisolone-equivalent) at baseline, mg/day | 45.0 (35.0–50.0) | 40.0 (40.0–40.0) | 0.762 |
| Year in which remission induction therapy was initiated | | | |
| In 2021 or later | 0 (0) | 0 (0) | - |
| At 1 month |  |  |  |
| Daily GC dose (prednisolone-equivalent), mg/day | 20.0 (15.0–20.0) | 20.0 (20.0–20.0) | 1.000 |
| %Change in the daily GC dose from baseline to 1 month, % | −25.0 (−30.0–−10.0) | −20.0 (−20.0–−20.0) | 0.766 |
| At 3 months |  |  |  |
| Daily GC dose (prednisolone-equivalent) at 3 months, mg/day | 10.0 (10.0–12.5) | 9.0 (9.0–9.0) | 0.289 |
| %Change in the daily GC dose from baseline to 3 months, % | −31.0 (−40.0–−22.5) | −31.0 (−31.0–−22.5) | 1.000 |
| Cumulative GC dose (prednisolone-equivalent) up to 3 months, mg | 1935 (1668–2145) | 1618 (1618–1618) | 0.385 |

Data are presented as median (IQR) or as n (%), unless otherwise indicated. ANCA, anti-neutrophil cytoplasmic antibody; PR3, anti-proteinase 3; MPO, anti-myeloperoxidase; GPA, granulomatosis with polyangiitis; MPA, microscopic polyangiitis; BVAS, Birmingham Vasculitis Activity Score; VDI, Vasculitis Damage Index; RTX, rituximab; CY, cyclophosphamide; GC, glucocorticoid

For statistical analyses, **p* < 0.05, ***p* < 0.01. *P*-value: Wilcoxon rank sum test, Fisher’s exact test

† Organ involvement was based on BVAS ≥ 1.

‡ An infusion of methylprednisolone for 3 consecutive days at a dose of 500 or 1000 mg per day.
